# Exploring Entrepreneurial Intention and Student Engagement of Youth Living in Poverty

**DOI:** 10.3390/bs14110995

**Published:** 2024-10-25

**Authors:** Rasha Mahmoud Khodor, Oliver Valero Coppin, Isabel Alvarez Canovas

**Affiliations:** 1 Department of Educational Theories and Social Pedagogy, Universitat Autònoma de Barcelona, 08193 Bellaterra, Spain; rashamahmoud.khodor@autonoma.cat; 2 Department of Mathematics, Universitat Autònoma de Barcelona, 08193 Bellaterra, Spain; oliver.valero@uab.cat

**Keywords:** youth living in poverty, entrepreneurial intention, student engagement, educational attainment, promotive factors

## Abstract

Graduating from secondary education for adolescents living in poverty is challenging. Strong entrepreneurial intention and student engagement among youth living in poverty often play a protective role in reducing school dropout and fostering school completion, which results in improved educational attainment. However, research on this topic is scarce. A total of 1135 adolescents took part in this cross-sectional study, 50.9% of which were females. On average, they were 16.4 years old. They were all upper secondary school students from ten public and private schools in Lebanon. They completed instruments measuring entrepreneurial intention and student engagement. This study explored the covariate associations between risk and promotive factors through four dimensions of entrepreneurial intention and two components of student engagement (cognitive and psychological engagement). It shows positive associations for entrepreneurial intention with both individual factors (age) and social factors (working mother and private school). Negative associations for student engagement were found in all (individual and social) factors with the exception of the father’s job, which did not present any association. The findings provide insight for policymaking to empower schools to promote school completion and educational attainment among these youth by providing policy initiatives and school-based interventions that target entrepreneurial exposure and engagement strengthening, hence meeting young people’s individual, family, and school community needs.

## 1. Introduction

Numerous papers have been published on the impact of poverty on young people’s school experiences and educational outcomes (e.g., school dropout and failure for educational attainment) [1,2]. Such studies claim that the consequences of poverty increase risks to youth healthy development and transition into adulthood [3,4]. However, few studies focus on how school experiences contribute to shaping the future educational goals and outcomes of young people living in poverty [5]. 

A plethora of studies have addressed the relationship between entrepreneurial intention (EI) and economic development on one hand [6,7] and student engagement (SE) and school dropout on the other [8,9,10]. Nonetheless, almost none has linked the two. Mostly, existing studies predominantly focus on the basic educational needs of young people, thus neglecting the nuanced needs of economically vulnerable youngsters [11]. Being responsive agents at the center of the increasingly interconnected and distal spheres of influence in the environment, such as family and school community, adolescents’ school experiences and educational outcomes are shaped by the complex interplay of reciprocal and interactive individual and social factors [12,13]. Given the history of negative educational experiences of youth living in poverty, nurturing adolescents’ EI and SE is important not only to support school completion and promote educational attainment, but also to foster their resilience and ability to cope amidst the challenges posed by poverty [14]. 

This cross-sectional study explores how EI and SE are associated with individual, family, and school community factors among adolescents in poverty attending formal upper secondary education—arguably the optimal stage for fostering entrepreneurship [15] and strengthening engagement [16]. The findings of this study stress the need to increase school completion and promote educational attainment among youth living in poverty by increasing promotive factors across their social environments. Providing a strength-based approach, this study aims to inform policy initiatives and school-based interventions to concurrently promote EI and SE among youth living in poverty—rather than focus on school failure—with the goal of promoting healthy transition from adolescence into early adulthood despite the challenges posed by poverty. 

In Lebanon, young people attending formal upper secondary education are among the vulnerable poor population with persistent disparities rising to 53.1% of the multidimensional poverty index (MPI). A recent value of youth unemployment peaked at 23.74%, and economic inequality ranks this Middle Eastern country at 129 out of 141 countries [17]. Hence, risks of underachievement and low engagement increase for such impoverished youth marking a point that threatens their future expectations and goals for school completion and educational attainment [18]. Formal upper secondary education rates (2022/2023) in Lebanon provide a stark example. Student enrolment indicated 12.4%, substantially below free compulsory primary education, which averages 83.5%. Highlighting a worrisome trend in school completion, transition rates (SDG4.1.4) showed a decrease by 2.5% on average, and the over-aged student percentage (SDG4.1.6) increased from 19.61% to 30.07% [19]. The lack of both EI and SE of these youth living in poverty, who are not exposed to any entrepreneurial experience in formal upper secondary education, can be associated with detrimental effects. Helping them concurrently develop their entrepreneurial intention and strengthen their engagement is crucial to reduce school dropout and non-completion. This necessitates promoting change towards school completion and educational attainment to help these youth cope with the adversities of poverty. 

### 1.1. Entrepreneurial Intention (EI) 

EI is considered the single best indicator of future entrepreneurial behavior [20], with its strength affecting the performance of entrepreneurial behavior [21]. This intention has been presented as a shared element in the relationship between the student and the entrepreneurial experience in various models and constructs [22]. Such an experience consists of interrelated and interactive cognitive dimensions within the student’s immediate environment (family and friends) and broader school environment (teachers, peers, and colleagues) [23], through which both students and their ecologies benefit [24]. This dominating cognitive approach to EI [25] considers three attitudinal and motivational determinants that reflect perceptions towards entrepreneurial behavior, namely, personal attitude (PA), perceived behavioral control (PBC), and subjective norm (SN). PA refers to the attitude, valuation, and affective and evaluative considerations towards a particular behavior. PBC focuses on an individual’s ability to perform and control that behavior, and SN refers to the degree of favorability of social support provided by essential relationships with significant people towards the behavior. EI can hence be understood as a variable aspect of the perceived school experience influenced by factors in the student’s cognitive ecologies [26].

### 1.2. Student Engagement (SE)

The multidimensional and malleable nature of the interactive relationships between the student and school experience is a common element in the numerous conceptualizations and constructs of SE [27,28,29]. These relationships are generally considered as encompassing many united interrelated components, including cognitive and psychological engagement, that vary in response to students’ developmental ecologies [30]. Cognitive and psychological engagement have less observed internal and alterable indicators that need to be studied to determine the degree of youth’s engagement with school. Cognitive engagement (CE) encompasses students’ thought processes that promote self-regulation, value and investment in learning, relevance of schoolwork for future endeavors, and personal goals and autonomy. Psychological engagement (PE) refers to students’ feelings of school identification, connectedness, belongingness, and supportive relationships with the family and school community. Therefore, SE can be considered a mutable aspect of the perceived subjective school experience, influenced by factors in the students’ psychological and cognitive ecologies [31,32]. 

### 1.3. Entrepreneurial Intention, Student Engagement, and Negative Educational Outcomes

Negative school experiences expose general youth population and particularly those living in poverty to a greater risk of negative short- and long-term educational outcomes [33]. EI has a highly correlated and mutually reinforcing U-shaped relationship [7] with the influence of economic [34] and wealth inequalities, which shape parents’ educational expectations and young people’s educational outcomes, directly predicting their future educational attainment [35]. Likewise, there is a high correlation and mutual reinforcement between school dropout and student disengagement [8]. The most cited factor of SE in research, especially throughout its most effective risk domain, is low socio-economic status [36]. The associated negative attitude towards school, underachievement, poor school connectedness and belongingness, lack of support from the family and school community, and low socio-economic status of families [37] lead to negative educational outcomes for young people living in poverty, such as school dropout and non-completion and failure for educational attainment [38]. 

### 1.4. Study Framework 

EI and SE are affected by both risk and promotive factors operating in young people’s social ecologies. Based on a risk and resilience framework [39], this study postulates that increasing promotive factors can decrease the influence of risk factors for youth living in poverty through the simultaneous development and strengthening of EI and SE. This framework is derived from the ecological theory [13]. It situates young people within dynamic social ecologies in which multi-systemic factors interact and influence individual, family, and school community experiences, psychological and cognitive development, EI, and SE. The central premise of the risk and resilience framework is that youth development occurs at an intersection of risk and promotive factors (including individual skills and external influences). Promotive factors, in the current context, include abilities and/or resources to support influential positive school experiences and outcomes, the value and relevance of school in promoting young people’s adaptation, and successful navigation between adverse experiences and risky outcomes [39]. Fostering and protecting adolescents’ healthy development and resilience against the adversities of poverty are also addressed [40]. 

### 1.5. Promotive Factors and Risk Factors for Entrepreneurial Intention

#### 1.5.1. Individual Factors

There is a determining relationship between EI and individual attitudinal motivational dimensions that often lead to entrepreneurial behavior [41] among both youth in the general population and those living in poverty. EI can be affected positively by individual factors. High levels of PBC strongly predict increased EI and foster student knowledge, experience, ability to engage in entrepreneurial behavior, and assess obstacles that may prevent it [42]. PA reflecting strong favorability and positive valuation towards entrepreneurial behavior is related to high EI. These constructs are affected by exogenous influences, such as culture and demographic variables, which, in their turn, affect EI [43]. These determinant antecedents and their socio-environmental stimuli influence the development of students’ EI and anticipatory behavior. This mitigates the effects of unemployment and social mobility of economically vulnerable youth [44].

Conversely, the development of EI can also be negatively influenced by individual constructs. An unfavorable personal attitude and negative valuation towards entrepreneurial behavior negatively affects EI. Low levels of PBC have an impact on students’ self-perceived efficacy and behavior control, thus decreasing EI and hindering the expected behavior [45]. These exogenous influences created by the social environment exert a social pressure that affects the change in student’s perception and attitude towards entrepreneurial behavior [46].

#### 1.5.2. Social Factors

EI is not solely influenced by individual factors. It is co-produced by relationships operating in the student’s social ecologies affected by diversified cultural values and normative beliefs [6]. Student perception of the high ability and social support of significant role models (family, peers, and colleagues) towards entrepreneurial behavior might increase EI and facilitate the intended behavior [47].

However, student perception of negative or opposing social pressure could negatively affect EI and hinder entrepreneurial behavior. The joint influences of social support, individual attitude, and agency towards the behavior shape intention, choice, and implementation [48].

The development of student EI fosters a favorable perception of the relevance of schoolwork for meeting achievement needs and future outcomes. It is hence motivational and promotive for dropout prevention. As a result, it increases school completion and educational attainment of young people in general and vulnerable young people in particular [49]. Fostering the development of young people’s EI can be perceived as their response and perception of the underlying processes of recognizing an opportunity for entrepreneurial behavior within the developing environment [50]. It contributes to the establishment of their future-oriented behavioral outcomes of goal setting and achievement [51].

### 1.6. Promotive and Risk Factors of Student Engagement

#### 1.6.1. Individual Factors

There is a strong relationship between negative school perceptions and beliefs about school and lack of SE [52] among young people in the general population and, in particular, among the economically vulnerable. This negativity is related to underachievement and is strongly associated with the irrelevance of schoolwork for future goals, thus diminishing the meaning and value of school [53]. They generate thoughts of unfavorableness of school and decrease of adolescents’ involvement, agency, and investment in school-related areas [54]. Negativity towards school is interrelated with decreased autonomy and self-regulation, leading to poor investment in learning and preventing future goal orientation [55]. This critically impacts engagement and increases the likelihood of dropping out of school [56]. The lack of positive perceptions towards school also affects students’ school satisfaction and ability to establish positive relationships with teachers and peers in the school community, which are crucial for SE [57]. In the absence of strong support, vulnerable young people experience negative school outcomes, such as dropping out of school, which is largely associated with low school completion and school failure [58].

In contrast, having positive perceptions and beliefs about school is an important promotive factor for increasing engagement and reducing dropout [59]. Such perceptions and beliefs are linked to strong self-regulation, autonomy, and school relevance for future goals that increase the student ability to engage in learning. The dispositions of an engaged student encompass thriving educational endeavors and positive school identification with strong school connectedness, belongingness [60], high motivation, and the perception of schoolwork as relevant for future-oriented goals and aspirations [61]. This reflects high self-efficacy for self-regulation correlating positively with motivation, valuing of school, effective investment in learning, and future goal setting, which increases the student’s ability to promote the subjective value of learning and achievement [62]. The likelihood of dropping out of school and non-completion are hence reduced, and educational attainment is encouraged.

#### 1.6.2. Family and School Community Factors

SE is not driven and affected solely by individual factors. It is co-constructed [63] by the relationships operating within students’ social ecologies [64]. Perceived positive social support for learning from the family and school community is linked to high SE profiles [65]. Levels of learning support provided by parents, school problem solving, and effective use of appropriate rewards to increase student motivation [66] are vital for reducing school dropout risk. They predict school connectedness, belongingness [67], and receptiveness leading to improved school value and investment in learning [68], thus contributing to school completion and educational attainment. Likewise, school community engagement strengthens young people’s identification with and connectedness to school and provides ongoing supportive relationships that foster SE. Affective and supportive teacher–student relationships provide students with social support for learning [69]. Positive and supportive relationships with peers in general, especially peer groups, are vital for students’ socialization and the shaping of their future educational aspirations and achievements. They are likely to affect young people’s overall school experiences, outcomes, and life trajectories [70].

Conversely, poor social support is related to a lack of SE. Families with economic difficulties provide less or no support and involvement in their children’s learning [71]. Negative school community engagement, such as conflictual student–teacher relationships and lack of peer acceptance or support, devalues school and is related to the breakdown of positive social bonding and support, which often co-occur with student disengagement [72]. It instigates negative trajectories for school non-completion and dropout [1]. Young people who receive effective family support and are proactively engaged in their school community with reciprocal social resources and support are less likely to disengage or drop out of school. They have greater opportunities for educational attainment [73].

### 1.7. The Current Study

This study aimed to broaden the understanding of EI and SE among adolescents attending formal upper secondary education and living in poverty. It also sought to assess how entrepreneurial intention and psychological and cognitive engagement are deeply related to the factors processing in the individual, parental, and school community systems of these youth. Researchers explored individual values (age and gender), parents (parent’s occupation) and school community (type of school). The multilevel ecological approach showed relevant supportive factors that constitute key indicators of EI and SE for youth in general and those living in poverty in particular. Thus, this study provides grounds for both policy initiatives, reforms, and recommendations and school-based interventions to enhance young students’ individual and community endurance and ultimately increase their school completion and educational attainment.

The aim of this study is double fold. First, it seeks to explore and examine entrepreneurial intention, EI, and cognitive and psychological engagement, PE, and their related factors and the influencing demographic characteristics of youth attending formal secondary education and living in poverty. The second is to highlight the effect of the concurrent nurturing of youth’s EI and SE for the betterment of school experiences and educational outcomes, leading to enhanced school completion and educational attainment. Such enhancement contributes to youth coping with poverty adversities and healthy transition to adulthood.

## 2. Materials and Methods

### 2.1. Sample and Procedure

This study is based on a sample of (*N* = 1135) 10th, 11th, and 12th grade upper secondary school students from five public and five private schools located in Beirut and Mount Lebanon. Of the participating students, 50.9% were females. On average, they were 16.4 years old. The necessary consents were obtained. The students also gave their active consent to participate and replied to the questionnaires in pen and paper. In fall 2023, the students responded to two self-reporting questionnaires on EI and SE within approximately 30 min. During data collection, each classroom was supervised by a trained research assistant. This project was approved by the Research Ethics Committee of the Universitat Autònoma de Barcelona and the Lebanese Ministry of Education and Higher Education.

### 2.2. Measures

#### 2.2.1. Entrepreneurial Intention Questionnaire (EIQ)

EI, as perceived by secondary students in Lebanese schools, was measured using 4 indicators [43]: personal attitude (PA) (five items; α = 0.871; e.g., “Being an entrepreneur implies more advantages than disadvantages to me” (range = 1–7, M = 5.47, SD = 1.26), subjective norm (SN) (two items; α = 0.694; e.g., “If you decided to create a firm/company, would people in your close environment approve of that decision?” (range = 1–7. M = 5.55, SD = 1.16), perceived behavioral control (PBC) (six items; α = 0.857; e.g., “To start a business/firm/company and keep it working would be easy for me” (range = 1–7. M = 4.53, SD = 1.27), and entrepreneurial intention (EI) (five items; α = 0.694; e.g., “I will make every effort to start and run my own firm/company” (range = 1–7. M = 4.94, SD = 1.48). Each item was rated on a seven-point scale ranging from (1) strongly disagree to (7) strongly agree.

#### 2.2.2. Student Engagement Instrument (SEI)

SE, as perceived by secondary students in Lebanese schools, was measured using two dimensions of the student engagement instrument [31]: psychological engagement (PE) (nineteen items; α = 0.874; e.g., “Overall, adults at my school treat students fairly”) and cognitive engagement (CE) (sixteen items; α = 0.809; e.g., “The tests in my classes do a good job of measuring what I am able to do”). Each item was rated on a four-point scale ranging from (1) strongly disagree to (4) strongly agree.

PE was estimated via young people’s perception of the values of the teacher–student relationship (TSR), peer support for learning (PSL), and family support for learning (FSL). TSR (range = 1–4. M = 2.18, SD = 0.53) was a 4-point ordinal scale with response options ranging from (1) strongly disagree to (4) strongly agree. PSL (range = 1–4. M = 2.07, SD = 0.58) was a 1–4-point ordinal scale with response options ranging from (1) strongly disagree to (4) strongly agree. FSL (range = 1–4. M = 1.69, SD = 0.62) was a 1–4 ordinal scale with response options ranging from (1) strongly disagree to (4) strongly agree.

CE was estimated by young people’s perception of the value of control and relevance of schoolwork (CRSW), future aspirations and goals (FG), and extrinsic motivation (EM). CRSW (range = 1–4. M = 2.26, SD = 0.54) was a 4-point ordinal scale with response options ranging from (1) strongly disagree to (4) strongly agree. FG (range = 1–4, M = 1.68, SD = 0.58) was a 4-point ordinal scale with response options ranging from (1) strongly disagree to (4) strongly agree. EM (range = 1–4, M = 3.13, SD = 0.83) was a 4-point ordinal scale with response options ranging from (1) strongly disagree to (4) strongly agree.

### 2.3. Age

Age was a single-item covariate that measured young people’s age in years. Ages ranged from 14 to 18. The average age of the sample was 16.4 (see Table 1).

### 2.4. Gender

Gender was a single-item covariate that dichotomously identified youth as male (49.1%) or female (50.9%).

### 2.5. School Type

The school type was a single-item covariate that dichotomously identified public (53%) or private school (47%).

### 2.6. Parents’ Occupational Status

The parents’ occupational status was a single-item covariate that dichotomously identified mothers as employed (43.6%) or unemployed (56.4%) and fathers as employed (91.1%) or unemployed (8.9%).

### 2.7. Statistical Analysis

For the EI and SE questionnaires, bivariate analyses were performed between the students’ socio-demographic characteristics. Then, structural equation modelling (SEM) was used to test a 4-factor model for the EI and a 3 + 3 factor model for the SE within a multiple indicators multiple causes (MIMIC) framework. Due to the non-normality of the items, they were considered as ordinal data based on the polychoric correlation matrix. The MIMIC approach within SEM [74] consists of two steps: (1) fitting a confirmatory factor analysis (CFA), in which latent variables are predicted by observable variables, and (2) fitting a structural model to investigate the relationships between directly measured covariates and latent factors [75]. The covariates used in the model were gender, age, school type, and mother’s and father’s job.

The adequacy of model fit in each model was assessed using established SEM cut-off criteria: comparative fit index (CFI) and Tucker–Lewis index (TLI) greater than 0.95, root mean square error of approximation (RMSEA), and standardized root mean square residual (SRMR) less than 0.06 [76]. Internal consistency was assessed using the omega 3 coefficient [77].

Statistical analysis was performed with R version 4.3.2. Models were estimated using the Lavaan package [78].

## 3. Results

### 3.1. Correlation between Entrepreneurial Intention and Student Engagement Factors

The four EI factors (PA, PBC, EI, and SN) were significantly positively correlated with covariates theorized to promote EI: age (PBC), private school (SN), mother’s job (PA and SN), and father’s job (SN). They were significantly negatively correlated with covariates to promote EI: male (SN), private school (EI), and mother’s job (EI) (see Table 2).

The six SE factors (TSR, PSL, FSL, CRSW, FG, and EM) were significantly positively correlated with covariates theorized to promote SE. The strong associations were male and age (CRSW and FG) and private school (EM), and those significantly negatively correlated with covariates theorized to promote SE were private school (TSR, PSL, FSL, CRSW, FG), mother’s job (TSR), and father’s job (TSR) (see Table 3).

### 3.2. Measurement Model

Both confirmatory factor analyses (EI and SE) assessed the adequacy of the three-factor measurement model before adding covariates.

**Figure 1:**
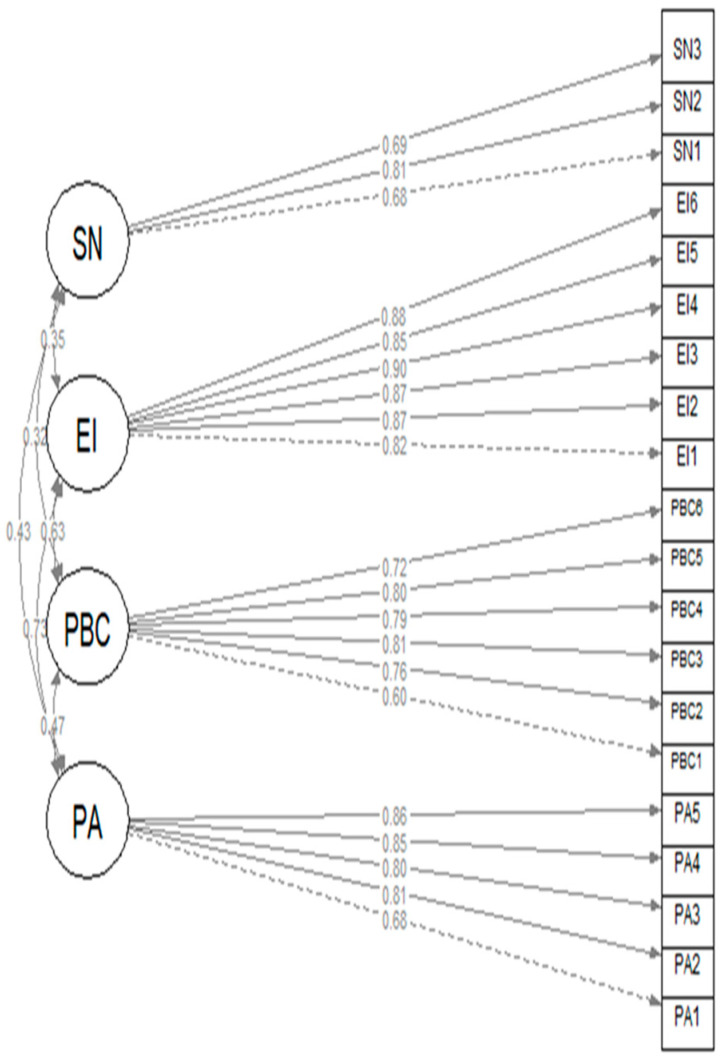
Measurement model: confirmatory factor analysis of four dimensions of EI.

The EI model presented a good fit to the data (χ2. = 690, df = 164, *p* < 0.001; RMSEA = 0.053 [0.049, 0.057]; CFI = 0.995; TLI = 0.995; SRMR = 0.038). The standardized factor loadings are presented in Figure 1. Omega3 values were 0.88, 0.88, 0.95, and 0.73 for PA, PBC, EI, and SN, respectively.

**Figure 2:**
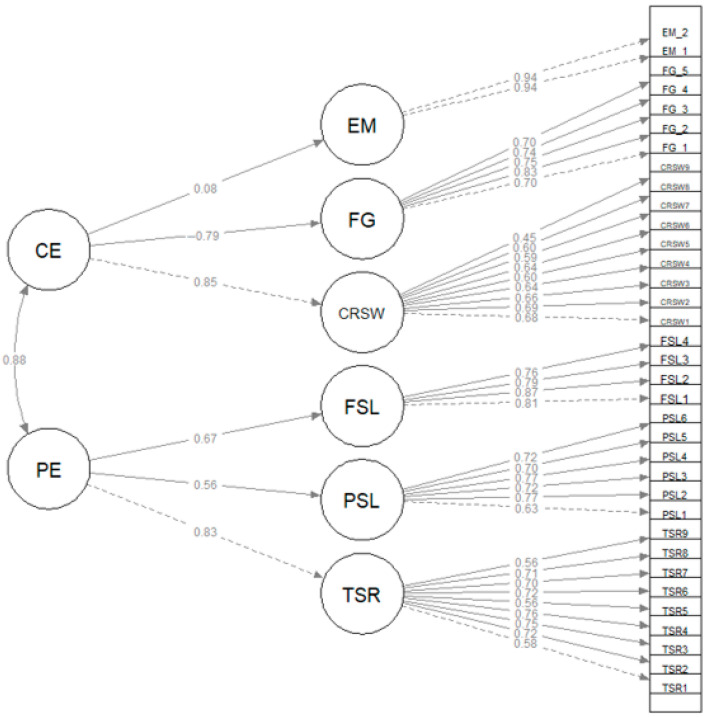
Measurement model: confirmatory factor analysis of two dimensions of SE.

The SE model also presented a good fit to the data (χ2. = 2347, df = 554, *p* < 0.001; RMSEA = 0.053 [0.051, 0.056]; CFI = 0.972; TLI = 0.969; SRMR = 0.064). The standardized factor loadings are presented in Figure 2. Omega3 values were 0.86, 0.83, 0.83, 0.85, 0.71, and 0.79 for TSR, PSL, FSL, CRSW, FG, and EM, respectively.

Internal consistency (value > 0.7) indicates consistency in higher reliability.

### 3.3. Structural Models

#### 3.3.1. Model 1: Entrepreneurial Intention (EI)

In EI model 1 (Table 3), gender, age, school type, and mother and father’s jobs were added as covariates. The EI model showed a good fit (χ2. = 2416, df = 709, *p* < 0.001, RMSEA = 0.049, CFI = 0.970, TLI = 0.967, SRMR = 0.062). The inclusion of individual-level covariates had no discernible impact on the factor loadings of the latent variable indicators.

Male gender had a statistically significant association with SN (b = −0.216) (see Table 4). Being male is associated with lower SN. This means male students perceive less social pressure or approval from others regarding EI than female students. This could reflect gender norms that influence male students to rely less on the opinions of others when making entrepreneurial decisions.

Age had a positive association with PBC (*b* = 0.197). Older students tend to have higher perceived behavior control. As students get older, they feel more able to control their behaviors and actions, which is essential for entrepreneurial activities. This could be due to greater experience, maturity, and confidence that come with age.

The strongest association was found for both private school and EI (*b* = −0.290) and SN (*b* = 0.240). Students in private schools tend to have lower EI than those in public schools. This is possibly due to different career expectations, values, aspirations, and educational focus. Attending a private school has a highly positive impact on subjective norms. Students in such schools are more likely to perceive subjective pressure or approval towards certain behaviors.

The mother’s job had a significant association with PA (*b* = 0.225), as having a working mother is positively associated with students’ personal attitude towards EI. This might be because seeing their mother in a professional role could inspire a positive attitude towards achieving their own entrepreneurial goals. Despite having a positive influence on personal attitude, a working mother is associated with lower EI in her children. This might reflect the complexities and challenges working mothers face. They may deter their children from pursuing entrepreneurial paths that may appear risky or uncertain EI (*b* = −0.236). Students with working mothers perceived greater social approval regarding entrepreneurial activities. This could indicate that these students perceived their mothers as role models who support entrepreneurial behavior (SN (*b* = 0.184)).

Finally, the father’s job had an association with SN (*b* = 0.226). Like mothers, having a working father is associated with higher subjective norms. This suggests that working fathers also influence their children’s perception of social approval or pressure towards EI.

#### 3.3.2. Model 2: Student Engagement (SE)

The SE model (Table 5 and Table 6) showed a good fit (χ2. = 976, df = 254, *p* < 0.001, RMSEA = 0.050, CFI = 0.994, TLI = 0.992, SRMR = 0.051). The inclusion of individual-level covariates had no relevant impact on the factor loadings of the indicators of the latent variables.

The results for psychological engagement (PE) (see Table 5) were as follows:

Male gender had a statistically significant association with TSR (*b* = −0.252). This means male students have a less favorable teacher–student relationship due to various factors, such as different communication styles, classroom engagement levels, or societal expectations and PSL (*b* = −0.227). It shows male students have less peer support for learning, and this could be due to social dynamics where male students may prioritize other activities over academic collaboration.

Age had an association with PSL and was highly significant (*b* = −0.261), suggesting that older students tend to have less PSL. As students get older, they may become more self-sufficient or face greater academic competitiveness, which reduces collaborative learning.

The strongest association was private school with TSR and was highly significant (*b* = −0.811). Attending a private school is strongly associated with a weaker teacher–student relationship. This might reflect different teaching styles, class sizes, student expectations, or school policies.

The mother’s job had a significant association with TSR (−0.269). The fact that the mother works is associated with a weaker teacher–student relationship. This could be due to less parental involvement in school activities, which could affect the adolescent’s interaction and engagement with teachers.

The exception was FSL, which did not have an association with any of the covariates. The father’s job did not have an association with any of the covariates.

The results for cognitive engagement (CE) (see Table 6) were the following:

Gender had an association with FG (*b* = −0.393). Being male is associated with weak future goals. Male students might have lower aspirations or less clarity about their future academic or career goals compared to female students.

Age was associated with FG (*b* = −0.459). Older students tend to have significantly lower future goals. This might suggest uncertainty or changing priorities as students get older. Similarly, in EM (*b* = 0.164), older students tend to have significantly higher extrinsic motivation. This might be due to greater pressure or to performing well academically and preparing for future opportunities as students get older.

Private school was associated with CRSW (*b* = 0.552). Attending a private school is strongly associated with a higher perception of control and relevance of schoolwork. This may be due to factors such as personalized learning environments, smaller class sizes, and greater emphasis on SE in private schools. The strongest association was private school and FG (*b* = 0.634), indicating that students from private institutions might focus more on academic and career planning. Private school was also associated with EM (*b* = −0.362). Attending a private school is strongly associated with lower extrinsic motivation. This could reflect a greater emphasis on intrinsic motivation and personal satisfaction in private school environments.

The mother’s job had a significant association with CRSW (*b* = 0.503). Having a working mother is strongly associated with a higher perception of control and relevance of schoolwork. This could reflect greater independence and modelling of a strong work ethic. The mother’s job was also associated with FG (*b* = 0.477). Having a working mother is associated with higher future goals. This could be due to the modelling of a strong ethic and career focus and EM (*b* = −0.238). Having a working mother is associated with lower extrinsic motivation. This might be due to the development of intrinsic motivation and independence in children whose mothers work. The exception was the father’s job, which did not have an association with any of the covariates.

## 4. Discussion

The multilevel (personal, parental, and school community) framework is needed for addressing both EI and SE for youth attending upper formal secondary education and living in poverty in Lebanon. Stable household incomes, supportive school communities, and increasing favorable personal perspectives were associated with stronger EI and SE in the Lebanese context. Findings show the need for strengthening positive influences both at the personal and community levels for this young population struggling with poverty issues.

### 4.1. Individual Influences

#### Age and Gender

Some demographic variables sustained certain associations described in the results. For instance, identifying as male or being of a younger age had significant negative associations with both psychological and cognitive engagement, jeopardizing both the student–teacher relationship and peer support for learning and future goals. Such association may result from a decrease in their learning interest, especially when they consider that the curriculum is irrelevant to their future endeavors. Peer influence might create a pressure affecting their school experiences, especially at the adolescent stage. In addition, the lack of male role models in the family or school environment can be relatively influential [9,79]. Creating a supportive school environment for these young males that considers mentoring programs allows them to have positive relationships and interactions within their school community (teachers and peers). Furthermore, providing entrepreneurial exposure that targets school relevance for future goals might contribute to improving their engagement. These findings should motivate interventions that address gender discrimination policies, especially those related to women’s education [80], and their effect on women’s educational outcomes, school completion, and gender differences in coping and engagement [81]. How this might affect poverty, especially of young people from a low economic background, should be explored [82], as this is particularly relevant to studies promoting gender and peer relationships. The results also show how, as students get older, they have a negative association with peer support for learning [83] and future goals for academic life.

For EI, there were some positive associations with age and PBC and negative associations with male gender and SN. Discrepancies are observed in gender regarding attitudes to entrepreneurship [84]. Entrepreneurial passion and tendency for males show a stronger predictor of EI with an essential role of the cultural context [85].

The findings of this study stress the critical need for continuing professional development of teachers to devise strategies to better engage male students and enhance relationships with them [86]. Peer collaboration programs that foster peer support to improve a culture of collaborative learning are required. Age-appropriate support through the implementation of age-specific engagement strategies to maintain interest and collaboration among older students is also necessary. With respect to mentorship opportunities, older students should be paired with younger students in those programs. It is necessary to design gender-sensitive courses to promote an inclusive curriculum, ensuring that content appeals equally to young women and men pursuing their entrepreneurial careers.

### 4.2. Social Influences

#### Mother’s/Father’s Jobs and Family Support for Learning

Some covariates showed negative associations with the mother’s job for TSR and EM, affecting both psychological and cognitive engagement. This could be related to the mothers’ lack of time to be more involved in school matters. This, in turn, could lead to a lack of interaction and education levels and these students building up more intrinsic motivation and independent decision-making [87]. Mothers’ involvement in school declines substantially in secondary school. This decline can stem from a lack of parental economic resources and working status affecting a student’s engagement [79], educational goals, aspirations, and attainment and career trajectories [88]. Furthermore, mothers present more significant role models to their young daughters, but they exert an influence on the educational abilities of both daughters and sons to a certain degree [89].

In contrast, the positive association with the mother’s job had a positive impact on CRSW and FG. It is also worth noting that having a working father did not present any association for SE.

EI had positive associations with the mother’s job for students’ PA and SN, as mothers are role models for their children and influence student performance. It is worth mentioning that the father’s job also had a positive association with SN. Negative associations were found for the mother’s job and EI. Studies show that parents’ job satisfaction, including mothers, can considerably affect their children’s EI [90].

In this regard, the creation of programs involving parents, especially working mothers and fathers, could be important. For instance, parents could share their work experiences, discussing the value of entrepreneurship, and they could actively participate in school projects [91].

### 4.3. School Type

#### 4.3.1. Promoting Public Entrepreneurial Intention and Student Engagement

Student engagement in relation to the school type shows that attending a private school has a positive impact on CRSW, which may be related to having access to more resources than at public schools. For instance, personalized learning environments and private schools tend to pay more attention to academic and career planning. Public schools should also consider introducing extra-curricular activities focusing on EI to make students concentrate more on their academic performance. Formal and informal strategies might include flexible or alternative school-wide programs that support impoverished youth with meaningful and relevant learning and help them engage with the school community [92].

Regarding entrepreneurial intention, the results show a negative association in comparison to public schools. However, private schools show a highly positive impact on subjective norms, subjective pressure, or approval of certain behaviors. Public schools should consider introducing extra-curricular activities focused on EI to get students more involved in their educational attainment and concentrate on experiential learning that makes them more focused on achievement. Such activities allow them to better explore and exploit entrepreneurial opportunities [93]. School-based entrepreneurial interventions and programs (labs, exercises, and lectures) and career guidance [94] influence students’ appreciation and critical awareness towards entrepreneurship [95].

#### 4.3.2. The Key Role of Schools

Youth living in poverty are less likely to graduate from secondary education than their peers [96]. There is great potential for policymaking to empower schools to promote school completion and educational attainment among such adolescents. Policy initiatives and school-based interventions that focus on entrepreneurial exposure and engagement strengthening could be provided. Hence, the young people’s individual, family, and school community needs would be met. It is necessary to increase EI [97] and SE among youth living in poverty, as research suggests they often play a protective role in reducing dropout. This can be done by encouraging school completion, which results in improved educational attainment [98]. Completing secondary education represents an essential individual and social milestone. Without it, these young adults’ opportunities for surviving poverty are severely limited, and the risk of negative experiences and outcomes increases substantially [4].

Collaborative and supportive structures are needed for economically impoverished young people and their families to help redirect negative educational trajectories. The lack of EI and low levels of SE among youth living in poverty suggest that schools need to do more to better support vulnerable youth and their families [99]. It is crucial to support young people and families beyond the traditional school setting. School-based initiatives and family and community partnerships provide individual and social support and resources [100] and associate with increasing levels of student engagement [101], which could help redirect and limit the development of unfavorable entrepreneurial attitudes and low SE levels that inhibit school completion and are associated with school failure. Schools are an important place for individual and social development, including psychological and cognitive development during adolescence. They are uniquely positioned to detect risk factors and enhance promotive factors.

The findings of the study suggest that, for youth living in poverty, school experiences do not offer sufficient support. These results can inform policymaking to prioritize policy initiatives and designs through which school staff can identify risk factors and direct interventions to vulnerable youth and their families. This study underlines the critical need to focus attention on the concurrent development and strengthening of EI and SE among youth in poverty and on the important contribution of promotive factors to increase intentions and engagement despite the presence of substantial risk factors among the adversities and hardships of poverty.

### 4.4. Implications

This is a novel study that simultaneously explores the development of EI and the strengthening of SE, together with the individual and social factors of poor adolescents. By proposing the analysis from an ecological perspective, this paper provides a comprehensive reflection to envisage future policy initiation for school-based interventions. This reflection suggests that initiating policies for entrepreneurial exposure and engagement for the young population in formal secondary education in general, and in particular for vulnerable youth, should be considered as an essential policy agenda. Secondary education is the right time to initiate policies that strike a balance in fostering EI and ultimately increase school completion and promote educational attainment. By considering school completion and educational attainment as priority goals, the current policy agenda should focus on formal education in secondary schools, especially public ones. It should be extended to family and social ecologies that exert a powerful influence on students’ educational opportunities, interests, investment in learning, and future goals and aspirations [102]. Therefore, targeted initiatives and policy design for vulnerable youth living in poverty could effectively promote educational attainment and foster young people’s adaptive capacity and resilience amidst the adversities of poverty, thus facilitating and encouraging healthy development trajectories.

### 4.5. Limitations

Although this study has provided important data, several limitations should be noted. The data from this study are cross-sectional, which limits causal conclusions derived from the self-reports of upper secondary education students. Conducting a longitudinal study for a similar sample might provide a more comprehensive exploration of EI and SE. The research team, in collaboration with school staff, implemented practical strategies (such as providing students with guidelines and explanations about the importance of self-reporting their perceptions) to reduce threats of possible inaccurate and/or invalid responses of some young people to the questionnaires [103]. While the use of data collected through self-report questionnaires is often considered a practical strategy to help inform policies and interventions, it also has measurement limitations. Incorporating qualitative data collected through parent and teacher interviews might help diversify the results. There are other factors that affect EI and SE which this study does not take into account. Exploring additional contextual factors would be beneficial.

## 5. Conclusions

The concurrent development of EI and strengthening of SE among young people living in poverty is associated with reduced dropout and improved school completion and educational attainment. This study simultaneously explored the joint associations of EI and SE and individual, family, and social risk and promotive factors. It was found that EI determinants and (cognitive and psychological) SE components are linked to an interplay of mutable and risk factors operating in young people’s social ecologies. The results of the study provide an important contribution, as poor young people are a challenging population that has not been paid attention to in youth literature. This gap has limited the understanding of their complex educational needs. This study addresses this gap in literature by examining associations between factors within multiple levels of the experiences of youth living in poverty. It also offers insights into the important contribution that increasing promotive resources and support can make to help improve school completion and promote educational attainment. By exploring the associations and effects of these important factors on specific constructs theorized to understand EI and SE, this study provides added specificity to support the premise that higher levels of promotive factors among youth living in poverty develop EI and increase SE and can reduce risk factors and offset their potential. Schools in general, and upper secondary schools in particular, are powerful places where promotive factors in young people’s social environments can be increased. They can also mitigate risks by offering supportive resources. Promoting EI and SE concurrently is key to reducing negative outcomes and to redirecting the trajectories of poor young people towards a positive transition into early adulthood.

## Figures and Tables

**Table 1:** Demographic distribution of the sample by age, gender, school type, and parents’ occupational status.

Age (*N* = 1135)	
14–15	8.6%
15–16	31.1%
16–17	28.1%
17–18	28.1%
>18	4.1%
Gender (*N* = 1135)	
Male	49.1%
Female	50.9%
School Type (*N* = 1135)	
Public	53%
Private	47%
Parents’ occupational status (*N* = 1135) Mothers Employed Unemployed	43.6% 56.4%
Parents’ occupational status (*N* = 1135) Fathers Employed Unemployed	91.1% 8.9%

**Table 2:** Bivariate correlations between EI latent factors and covariates.

	2	3	4	5	6	7	8	9	10
1. PA	0.399 ***	0.614 ***	0.348 ***	0.794 ***	−0.006	0.0089	0.044	0.014	0.011
2. PBC		0.558 ***	0.243 ***	0.742 ***	−0.003	0.021	0.020	0.016	0.097 **
3. EI			0.265 ***	0.843 ***	−0.055	0.014	−0.001	−0.013	0.050
4. SN				0.592 ***	0.079	−0.080 **	0.078 **	0.058	0.041
5. EIQ					−0.001	−0.009	0.044	0.022	0.066 *
6. Private						0.016	0.258 ***	0.171 ***	−0.177 ***
7. Male							0.054	0.009	0.042
8. M_Job								0.004	−0.129 ***
9. F_Job									−0.061 *
10. Age									

PA personal attitude, PBC perceived behavioral control, EI entrepreneurial intention, SN subjective norm, EIQ entrepreneurial intention questionnaire, M_Job mother’s job, F_Job father’s job. * *p* ≤ 0.05; ** *p* ≤ 0.01; *** *p* ≤ 0.001.

**Table 3:** Bivariate correlations between SE latent factors and covariates.

	2.	3.	4.	5.	6.	7.	8.	9.	10.	11.	12.	13	14.
1. TSR	0.376 ***	0.413 ***	0.566 ***	0.440 ***	−0.089 **	0.755 ***	0.395 ***	0.688 ***	−0.316 ***	0.037	−0.086 **	−0.0633 *	0.023
2. PSL		0.383 ***	0.301 ***	0.328 ***	−0.016	0.763 ***	0.274 ***	0.624 ***	−0.120 ***	−0.029	−0.019	0.012	−0.058
3. FSL			0.369 ***	0.490 ***	−0.109 ***	0.793 ***	0.319 ***	0.669 ***	−0.152 ***	0.048	−0.054	−0.028	0.056
4. CRSW				0.515 ***	0.005	0.528 ***	0.683 ***	0.714 ***	−0.149 ***	0.121 ***	−0.003	−0.040	0.130 ***
5. FG					−0.108 ***	0.545 ***	0.623 ***	0.690 ***	−0.218 ***	0.194 ***	−0.064 *	−0.056	0.114 ***
6. EM						−0.093 **	0.616 ***	0.295 ***	0.162 ***	−0.011	0.064 *	0.038	−0.033
7. PE							0.424 ***	0.856 ***	−0.249 ***	0.025	−0.067 *	−0.033	0.010
8. CE								0.832 ***	−0.060 *	0.136 ***	0.011	−0.018	0.088 **
9. SE									−0.187 ***	0.093 **	−0.035	−0.031	0.057
10. Private										0.016	0.258 ***	0.171 ***	−0.177 ***
11. Male											0.054	0.009	0.042
12. M_Job												0.004	−0.129 ***
13. F_job													−0.061 *
14. Age													

TSR teacher and student relationship, PSL peer support for learning, FSL family support for learning, CRSW control and relevance of schoolwork, FG future aspirations and goals, EM extrinsic motivation, PE psychological engagement, CE cognitive engagement, SE student engagement, M_Job mother’s job, F_Job father’s job. * *p* ≤ 0.05; ** *p* ≤ 0.01; *** *p* ≤ 0.001.

**Table 4:** MIMIC estimates for association between EI and student characteristics.

	Personal Attitude		Perceived Behavior Control		Entrepreneurial Intention		Subjective Norm	
	*b*	*s.e.*	*b*	*s.e.*	*b*	*s.e.*	*b*	*s.e.*
Male	0.115	0.099	0.089	0.090	−0.027	0.087	−0.216 **	0.074
Age	−0.145	0.083	0.197 **	0.069	0.088	0.073	−0.020	0.058
Private	0.147	0.099	0.070	0.091	−0.290 ***	0.087	0.240 ***	0.074
Mother’s job	0.225 *	0.101	0.067	0.091	−0.236 **	0.088	0.184 *	0.077
Father’s job	0.106	0.126	0.084	0.122	−0.216	0.116	0.226 *	0.094

*b* unstandardized regression coefficient, *s.e.* standard error of the coefficient, EI entrepreneurial intention. * *p* ≤ 0.05; ** *p* ≤ 0.01; *** *p* ≤ 0.001.

**Table 5:** MIMIC estimates for association between SE and PE and student characteristics.

	Teacher–Student Relationship		Peer Support for Learning		Family Support for Learning	
	*b*	*s.e.*	*b*	*s.e.*	*b*	*s.e.*
Male	−0.252 *	0.114	−0.227 **	0.079	0.026	0.070
Age	−0.171	0.100	−0.261 ***	0.063	0.013	0.059
Private	−0.811 ***	0.107	0.133	0.075	0.063	0.069
Mother’s job	−0.269 *	0.117	0.083	0.081	−0.010	0.072
Father’s job	−0.257	0.153	0.200	0.113	0.042	0.096

*b* unstandardized regression coefficient, *s.e.* standard error of the coefficient, SE student engagement, PE psychological engagement. * *p* ≤ 0.05; ** *p* ≤ 0.01; *** *p* ≤ 0.001.

**Table 6:** MIMIC estimates for association between SE and CE and student characteristics.

	Control and Relevance of Schoolwork		Future Aspirations and Goals		Extrinsic Motivation	
	*b*	*s.e.*	*b*	*s.e.*	*b*	*s.e.*
Male	0.179	0.106	−0.393 **	0.124	−0.009	0.053
Age	0.043	0.083	−0.459 ***	0.099	0.164 ***	0.044
Private	0.552 ***	0.102	0.634 ***	0.124	−0.362 ***	0.050
Mother’s job	0.503 ***	0.108	0.477 ***	0.127	−0.238 ***	0.052
Father’s job	0.187	0.150	0.318	0.163	−0.124	0.073

*b* unstandardized regression coefficient, *s.e.* standard error of the coefficient, SE student engagement, CE cognitive engagement. ** *p* ≤ 0.01; *** *p* ≤ 0.001.

## Data Availability

The data of this manuscript will not be deposited. The datasets generated and/or analyzed during this study are not publicly available. The data that support the findings of this study were collected by the researcher and were strictly used for research purposes in this study.
